# Emergence of New Non–Clonal Group 258 High-Risk Clones among *Klebsiella pneumoniae* Carbapenemase–Producing *K. pneumoniae* Isolates, France

**DOI:** 10.3201/eid2606.191517

**Published:** 2020-06

**Authors:** Rémy A. Bonnin, Agnès B. Jousset, Adriana Chiarelli, Cécile Emeraud, Philippe Glaser, Thierry Naas, Laurent Dortet

**Affiliations:** Institut Pasteur–Assistance Publique/Hôpitaux de Paris–University Paris Sud, Paris, France (R.A. Bonnin, A.B. Jousset, A. Chiarelli, C. Emeraud, T. Naas, L. Dortet);; Faculty of Medicine University Paris-Sud, University Paris–Saclay, Le Kremlin-Bicêtre, France (R.A. Bonnin, A.B. Jousset, C. Emeraud, T. Naas, L. Dortet);; National Reference Center for Antibiotic Resistance: Carbapenemase Producing Enterobacteriaceae, Le Kremlin-Bicêtre, (R.A. Bonnin, A.B. Jousset, C. Emeraud, T. Naas, L. Dortet);; Assistance Publique/Hôpitaux de Paris, Bicêtre Hospital, Le Kremlin-Bicêtre (A.B. Jousset, C. Emeraud, T. Naas, L. Dortet)

**Keywords:** *Klebsiella pneumoniae*, KPC, carbapenemase, ST307, ST147, epidemiology, whole-genome sequencing, bacteria, bacterial infections, antimicrobial resistance, nosocomial infections, France

## Abstract

The worldwide spread of *Klebsiella pneumoniae* carbapenemase–producing *Klebsiella pneumoniae* (KPC-*Kp*) isolates was reported to be caused by dissemination of 1 clonal complex (i.e., clonal group [CG] 258, which includes sequence types [STs] 258 and 512). We conducted whole-genome sequencing and epidemiologic analysis of all KPC-*Kp* isolates in France in 2018 and found that new successful high-risk clones of ST147, ST307, ST231, and ST383 are now the main drivers of *bla*_KPC_ genes. The *bla*_KPC_ genes were mostly carried by Tn*4401a* and Tn*4401d* structures and a new non–Tn*4401* element. Our epidemiologic investigations showed that the emergence of these non-CG258 KPC-*Kp* isolates in France was linked to dissemination of these clones from Portugal. Thus, KPC-*Kp* epidemiology has changed in Europe, at least in several non–KPC-endemic countries of western Europe, such as France and Portugal, where CG258 is not the most prevalent clone.

In *Klebsiella pneumoniae* bacteria, resistance to carbapenems results in 2 main mechanisms: the production of an extended spectrum β-lactamase or plasmid-borne cephalosporinase associated with a decrease in permeability of the outer membrane (especially through alteration of OmpK35 and OmpK36 porins), or the production of a carbapenemase (*1*,*2*). In France, these carbapenemases are Ambler’s class A KPC enzymes; class B metallo-β-lactamases of NDM-, VIM- and, to a lesser extent, IMP-type; and Ambler’s class D oxacillinases of OXA-48–like type (*3*,*4*).

*K. pneumoniae* carbapenemase (KPC) was first identified in United States in the early 2000s (*5*). Since then, this carbapenemase has spread and has become endemic in several countries, including the United States, Israel, Greece, China, and Italy. It has also been sporadically described in many countries of Europe (*1*). The worldwide spread of KPC has been linked to the dissemination of a main clone of *K. pneumoniae* (sequence type [ST] 258) and a single-locus variant (ST512) (*6*). In Asia (especially China), ST11, another single-locus variant of ST258, is mostly reported among *bla*_KPC_-harboring *K. pneumoniae* isolates (*7*). In addition, a recently published study, conducted by the EUSCAPE working group in 2013 in Europe, revealed that the spread of carbapenemase-producing *K. pneumoniae* was driven by only a few clones (*8*). The most prevalent carbapenemase was KPC (45.5% [311/684 isolates]), and 72.7% (229/311) of KPC-producing *K. pneumoniae* (KPC-*Kp*) belong to the same clonal group (CG) 258, including ST258 and ST512. Whole-genome sequencing (WGS) analysis has suggested that ST258 and ST512 KPC-*Kp* spread out in Europe from 2 KPC-endemic countries: Greece (ST258) and Italy (ST512) (*6**,**9**–**11*). However, that study described the epidemiology of KPC in Europe in 2013, whereas the aim of our study was to describe the genomic characteristics of KPC isolates from a more recent period.

Analysis of the genetic context of *bla*_KPC_ has revealed that this gene is mostly localized into a class 2 transposon named Tn*4401* (*12*). Several variants of this Tn*4401* (Tn*4401a* through Tn*4401i*) have been reported with deletions upstream of *bla*_KPC_ within the promoter region (*13*,*14*). Consequently, expression of *bla*_KPC_ genes is complex and might involve different promoters, depending on the specific genetic environment and bacterial species. The 2 main promoters are named P1, which is in the vicinity of *bla*_KPC_, and P2, a hybrid promoter located partly in the inverted repeat right of IS*Kpn7* (*15*). In rare cases, *bla*_KPC_ genes have been described in genetic structure not related to Tn*4401* and are named non–Tn*4401* elements (NTE) (*16*). However, in NTE, the expression of *bla*_KPC_ is mediated by other promoters. Our study aimed to deeply characterize the epidemiology of KPC*-Kp* circulating in France in 2018.

## Material and Methods

### Strains Collections and Culture Conditions

We included all KPC-*Kp* sent to France’s National Reference Center for Antimicrobial Resistance during January 1–December 31, 2018. As previously described, we used isolates that were recovered from clinical and screening specimens and sent on a voluntary basis by any type of laboratory related to any health facility, such as private and public hospitals, nursing homes, and community laboratories (*3*,*4*). These laboratories were located throughout France, including overseas territories. KPC-*Kp* recovered by the National Reference Center for Antimicrobial Resistance represent ≈85%–90% of the KPC-*Kp* infection cases reported to the French Public Health Agency (R.A. Bonnin, L. Dortet, unpub. data). The collection used for WGS analysis represents a total of 63 nonduplicate isolates recovered from rectal screening (n = 45), urine (n = 12), blood cultures (n = 1), wound infections (n = 2), and respiratory samples (n = 2) and 1 isolate for which no recovery site information was available. Because the aim of the study was to evaluate the genetic diversity of KPC producers, we discarded from further analysis any duplicate isolates or isolates recovered from the same patient.

### Antimicrobial Susceptibility Testing and Carbapenemase Detection

We performed antimicrobial susceptibility testing by using the disc diffusion method on Mueller-Hinton agar (Bio-Rad, https://www.bio-rad.com) and interpreted results according to European Committee on Antimicrobial Susceptibility Testing guidelines as updated in 2018 (http://www.eucast.org). We determined MICs for colistin by using broth microdilution (Sensititer Thermofisher, https://www.thermofisher.com). We performed carbapenemase detection by using Rapidec Carba NP (bioMérieux, https://www.biomerieux.com), followed by immunochromatographic detection of the carbapenemase enzyme using NG-Carba5 test (NG Biotech, https://ngbiotech.com).

### WGS and Bioinformatic analysis

We sequenced all KPC-*Kp* isolates by using Illumina technology as previously described (*17*). We extracted total DNA from colonies by using the Ultraclean Microbial DNA Isolation Kit (MO BIO Laboratories, https://www.mobio.com) according to the manufacturer’s instructions. We prepared the DNA library as previously described (*17*) and performed de novo assembly and read mappings by using CLC Genomics Workbench 12.0 (QIAGEN, https://www.qiagen.com). We identified the acquired antimicrobial resistance genes by using Resfinder 3.1 (https://cge.cbs.dtu.dk/services/ResFinder/) and the CARD database (https://card.mcmaster.ca). We annotated the genomes by using RAST (*18*). We performed phylogenic analysis by using CSIphylogeny 1.4 (https://cge.cbs.dtu.dk/services/CSIPhylogeny) and visualized the genomes by using FigTree 1.4.3 (http://tree.bio.ed.ac.uk/software/figtree). We performed sequences alignments by using ClustalW (https://www.genome.jp/tools-bin/clustalw). We analyzed single-nucleotide polymorphisms (SNPs) on the whole genome by using CSIphylogeny V1.4 with parameters as follows: select minimum depth at SNP position at 10×, minimum distance between SNPs at 10 bp, and minimum SNP quality score of 30.

We constructed the genetic contexts by using de novo assembly or by mapping with reference genomes from GenBank and verified by in-house PCR as previously described (*17*). We analyzed plasmid contents of clinical isolates by using PlasmidFinder 2.1 to search for the replicase gene and by conducting manual searches for genes showing homology with the replicase gene.

## Results

### Low Prevalence of KPC Producers among Carbapenem-Resistant *K. pneumoniae* in France

In 2018, a total of 3,931 carbapenem-resistant *Enterobacteriaceae* were collected, including 1,259 carbapenem-resistant *K. pneumoniae*, among which 1,010 were carbapenemase producers. OXA-48-like enzymes were the most prevalent carbapenemases (69.4%), followed by NDM (17.1%); 37 isolates (3.7%) had a combination of both of these carbapenemases. KPC enzymes represent only 3.0% of all carbapenemases, corresponding to 6.8% (69 isolates, including 6 duplicated isolates) of all carbapenemase produced by *K. pneumoniae*. KPC also was produced by 13 non–*K. pneunomiae*, including 5 KPC-2 producers (2 *Escherichia coli*, 1 *Klebsiella oxytoca*, 1 *Enterobacter cloacae*, and 1 *Citrobacter koseri*) and 8 KPC-3 producers (5 *E. coli*, 1 *C. freundii*, 1 *E. cloacae*, and 1 *K. aerogenes*). Accordingly, *K. pneumoniae* is the most prevalent species (84.1%) among KPC producers.

### Antimicrobial-Susceptibility of KPC-*Kp*

**Figure 1:**
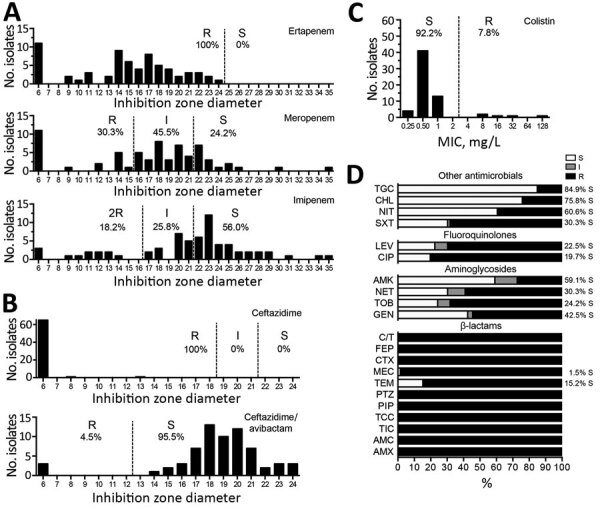
Susceptibility testing of *Klebsiella pneumoniae* carbapenemase–producing *K. pneumoniae* isolates, France, 2018. A) Antimicrobial susceptibility to carbapenems tested by using the disc diffusion method and interpreted according to European Committee on Antimicrobial Susceptibility Testing guidelines (http://www.eucast.org). B) Susceptibility to ceftazidime or ceftazidime/avibactam combination. C) MICs for colistin as determined by broth microdilution. D) Percentage of susceptibility to other antibiotic families. Where percentage of resistance is <100%, percentage of susceptible isolates is indicated. AMC, amoxicillin/clavulanate; AMK, amikacin; AMX, amoxicillin; CIP, ciprofloxacin; CHL, chloramphenicol; C/T, ceftolozane/tazobactam; CTX, cefotaxime; FEP, cefepime; GEN, gentamicin; I, intermediate; LEV, levofloxacin; MEC, mecillinam; NET, netilmicin; NIT, nitrofurane; PIP, piperacillin; PTZ, piperacillin/tazobactam; R, resistant; S, susceptible; TCC, ticarcillin/clavulanate; TEM, temocillin; TGC, tigecycline; TIC, ticarcillin.

Susceptibility testing revealed that all KPC-*Kp* were resistant to all broad-spectrum cephalosporins (ceftazidime, cefotaxime, cefepime) and monobactam (Figure 1). All KPC-*Kp* were resistant to ertapenem. According to European Committee on Antimicrobial Susceptibility Testing breakpoints, 62.1% (41/66) KPC-*Kp* isolates remained susceptible to imipenem and 30.3% (20/66) to meropenem (Figure 1). Ceftazidime/avibactam (98.5% susceptibility) and colistin (92.2%) remained the most potent agents (Figure 1, panels B and C). However, we identified 1 isolate resistant to ceftazidime/avibactam but susceptible to carbapenems (Figure 1). This isolate produces a new variant of KPC, named KPC-39, that has been reported to possess increased ceftazidime catalytic activity but also to have concomitantly lost its carbapenemase activity (*19*). Among other antimicrobial families, 84.9% KPC-*Kp* isolates were susceptible to tigecycline, 30.3% to sulfamethoxazole/trimethoprim, and 19.7% to ciprofloxacin (Figure 1, panel D). Resistance to aminoglycosides varied from 40.9% for amikacin to 75.8% for tobramycin.

### *bla*_KPC_ Variants and Associated Acquired Resistance Genes

**Figure 2:**
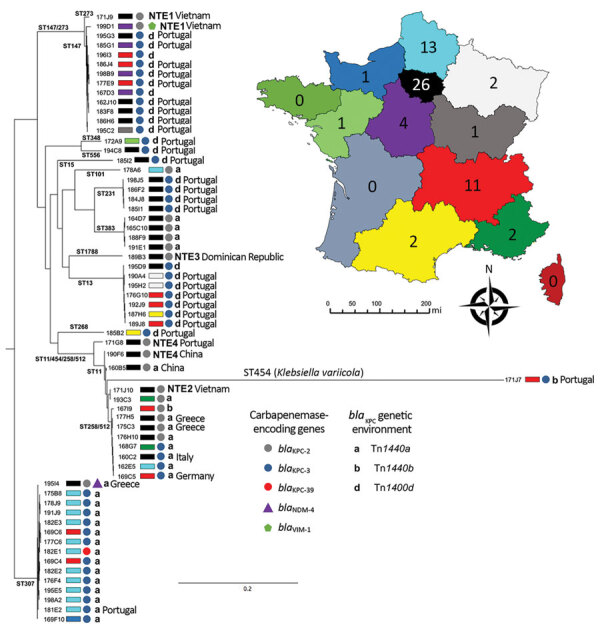
Phylogenetic analysis of *Klebsiella pneumoniae* carbapenemase–producing *K. pneumoniae* isolates, France, 2018. STs are indicated on the branches of the tree. Colored circles, triangles, or pentagons indicate carbapenemase type. Colored rectangles indicate region where isolates were recovered, as indicated on inset map; numbers on map indicate number of isolates. Genetic context indicated by isoform of Tn*4401* or NTE. Labels indicate links with foreign countries. Scale bar on tree indicates the number of single-nucleotide polymorpisms per position of common sequences. NTE, non-Tn*4401* element; ST, sequence type.

We performed WGS on 63 nonduplicate KPC-*Kp* isolates and identified their resistomes by using Illumina technology. In this collection, 44 isolates possessed the *bla*_KPC-3_ gene (69.8%), and 18 (28.5%) possessed the *bla*_KPC-2_ gene. One isolate harbored a novel single-nucleotide variant of *bla*_KPC-3_, *bla*_KPC-39_ (Figure 1, panel B). Two isolates produced 2 carbapenemases, including 1 isolate coharboring *bla*_KPC-2_ and *bla*_VIM-1_ and another 1 coharboring *bla*_KPC-2_ and *bla*_NDM-4_ (Figure 2). We identified additional antimicrobial-resistance determinants in all isolates (Appendix 1). Approximately 46% of the KPC-*Kp* isolates carried an extended spectrum β-lactamase encoding gene, including 22 isolates harboring *bla*_CTX-M-15_; 4 isolates coharboring *bla*_CTX-M-14_ and *bla*_CTX-M-15_; and 3 isolates with *bla*_CTX-M-14_, *bla*_CTX-M-3_, or *bla*_CTX-M-65_. The other acquired β-lactam resistance determinants encoded for the narrow-spectrum β-lactamases OXA-9 and TEM-1. To decipher quinolone resistance, we analyzed the presence of plasmid-mediated resistance determinants and known mutations in gyrase and topoisomerases (*20*–*22*). Resistance to quinolones was mediated by either mutation in gyrase *gyrA* affecting the residues S83 (p.S83I, n = 42; p.S83F, n = 7; and p.S83Y, n = 2) or D87 (p.D87N, n = 7; p.D87G, n = 3; and p.D87A, n = 2) or *parC* affecting the residues p.S80 (p.S80I, n = 51) or the production of *Qnr* (Qnr66-like, QnrB6, or QnrS1). Aminoglycoside resistance was caused by the production of the 16S RNA methylase RmtB (n = 7) or an aminoglycoside-modifying enzyme (encoding by *aac(3′)-IIa, aadA1, aadA2, aac(6′)-Ib-cr, or strA/strB*). Colistin resistance (n = 5) resulted systematically in chromosome-encoded resistance with alteration of the *mgrB* gene. In these isolates, 2 possessed a nonsense substitution (p.Q30*), 1 missense involved in colistin resistance (p.C27W) (*23*), an IS*Kpn26*-like inserted (with direct repeats [DRs] of 4 bp: TTAA), and a missense mutation leading to the disappearance of the start codon. No isolate had plasmid-encoded resistance *mcr*-1.

### Genetic Diversity of KPC-*Kp*

Phylogenetic analysis revealed that the 63 KPC-*Kp* belonged to 15 different clones (STs) circulating in France (Figure 2; Appendix 1). Although many studies have asserted that CG258 is responsible for the spread of *bla*_KPC_ (*6*,*8*–*11*), in our collection, only 8 isolates (12.7%) belonged to CG258 (4 each for ST258 and ST512). Furthermore, epidemiologic investigations revealed no link between these isolates (Figure 2). Because KPC is not widely disseminated in France, we did not expect to observe such clonal diversity. Indeed, the epidemiology of KPC in France is not comparable to what was reported in nearby countries in Europe where KPC is endemic, such as Greece and Italy, and where the spread of *bla*_KPC-2/-3_ is clearly linked to CG258 (*24*,*25*). Among the 8 isolates we identified that belonged to CG258, 3 were recovered from patients with travel history in Greece (isolate 175C3 and isolate 177H5) and Italy (isolate 160C2). In France, the 3 most prevalent clones are ST307 (with 15 isolates), ST147 (12 isolates), and ST13 (7 isolates). By using the 21 SNP cutoff value proposed by David et al. to identity a single hospital outbreak caused by a ST258 and ST512 cluster (*8*), we identified that the ST307 clone was overrepresented because of an outbreak that included 11 isolates (Figure 2; Appendix 2). However, this ST307 also included 4 isolates that were not related to this outbreak, such as the 195I4 strain, which was isolated from a patient who traveled in Crete (Greece) and possesses an additional carbapenemase-encoding gene (*bla*_NDM-4_). The second most prevalent clone, ST147, seemed to have disseminated upon distinct events (Appendix 2). Most of the ST147 isolates have been recovered from different areas with no epidemiologic link between the patients (Figure 2). A link with Portugal has been identified for most (9/11) patients infected or colonized with a KPC-3–producing *K. pneumoniae* of ST147 (Figure 2). The same link with Portugal was observed for 4 patients infected or colonized with a KPC-3–producing *K. pneumoniae* of ST231. Strains from ST383 represented a small outbreak for which cross contamination was evidenced (<20 SNPs between 4 isolates [Appendix 2]). One isolate (171J7) was distantly related to other clones and corresponded to *K. variicola*. KPC-2-producing *K. pneumoniae* isolates of ST11 were predominantly linked to patients who had a history of travel in Asia (China and Vietnam), where this ST is known to be the main vector of *bla*_KPC_ dissemination.

### Diversity of Genetic Vehicle Involved in Spread of *bla*_KPC_

**Figure 3:**
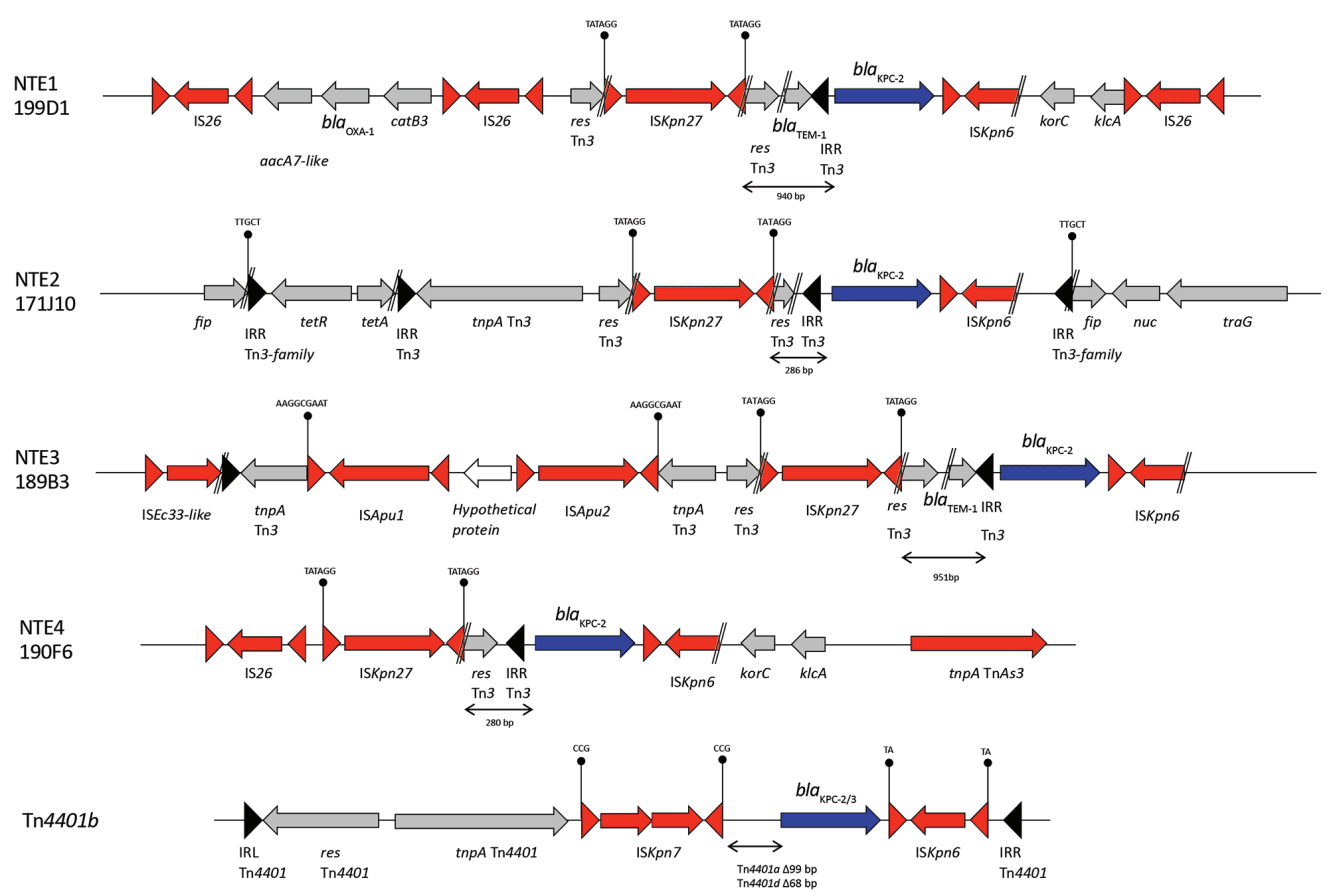
Analysis of genetic context of *bla*_KPC_ genes in *Klebsiella pneumoniae* carbapenemase–producing *K. pneumoniae* isolates, France, 2018. Different isoforms of NTE and Tn*4401* are represented. Inverted repeat sequences are indicated by triangles. Direct repeats are indicated by vertical lines. Genes are represented by arrows. NTE, non-Tn*4401* element.

Analysis of the close genetic context of *bla*_KPC_ highlighted diversity in the genetic structures at the origin of the acquisition of the carbapenemase-encoding gene. The well-known Tn*4401a* (in 29 isolates) and Tn*4401d* (in 26 isolates) were the most prevalent structures identified (Figure 2 and 3; Appendix 1). The KPC-*Kp* of the 2 main clones ST307 and ST147, *bla*_KPC_, is carried on Tn*4401a* in ST307 and Tn*4401d* in ST147. Two unrelated isolates (ST11–167I9 and the *K. variicola* 171J7 isolate) harbored *bla*_KPC_ in the Tn*4401b* isoform. In the remaining 6 isolates (ST273–171J9, ST147–199D1, ST1788–189B3, ST11–171G8, ST11–190F6, and ST11–171J10), *bla*_KPC-2_ is localized in an NTE element (Figures 2, 3). Although 3 isolates belonged to ST11, they displayed 200–800 SNPs of differences along their core genome, indicating that they were unrelated (Appendix 2). The links with 3 different countries (Portugal for ST11–171G8, China for ST11–190F6, and Vietnam for ST11–171J10) are consistent with this unrelatedness. Analysis of NTE elements revealed 4 different structures even if common features were observed (Figure 3). For instance, the presence of a fragment of IS*Kpn6* downstream of *bla*_KPC_ and a copy of IS*Kpn27* upstream were always present (Figure 3). DRs of TATAGG bracketing IS*Kpn27* indicated a transposition process that occurred inside the resolvase gene of Tn*3*. Immediately upstream of *bla*_KPC-2_ (74 bp), the presence of the inverted repeat right of Tn*3* is present in all NTE, indicating that all these structures were related. However, the NTE differed by the size of the deletions that are present between IS*Kpn27* and *bla*_KPC-2_ (from 280 bp in NTE-190F6 to 940 bp in NTE-199D1). We could observe a remnant of *bla*_TEM-1_ in longer structures, but it was not functional anymore. Analysis of the 4 NTE revealed that in NTE-199D1, several copies of IS*26* bracketed the whole structure, indicating that this IS might be involved in its acquisition by transposition or a recombination event. IS*26* has been recently demonstrated to be able to transpose and thus create a class I transposon by targeting another copy of IS*26* (*26*). NTE-171J10 is inserted in the *fip* gene of IncN-type plasmids with the presence of DRs surrounding the NTE-171J0 (Figure 3). The *fip* gene has already been demonstrated to be an integration hot spot in IncN-type plasmids (*27*,*28*). DRs as well as putative inverted repeats of Tn*3*-family transposon are present at the integration site (Figure 3). Moreover, the presence of the complete Tn*3* transposase gene indicated that NTE-171J10 might be functional. In NTE-189B3, a new class I transposon carrying a protein of unknown function has been identified. DRs bracketed IS*Apu1* and IS*Apu2*, indicating a transposition process mediated by these close insertion sequences (Figure 3).

## Discussion

In France, KPC producers (84.1% of *K. pneumoniae*) represent only 6.8% of all carbapenemase producers, far away from the global 72.7% found in Europe in 2013 (*8*). This relatively low prevalence of KPC producers in France compared with OXA-48–like and NDM producers has been reported since 2012 (*3*,*4*,*29*). We demonstrated unexpected clonal diversity among KPC-*Kp* isolated in France. A few overrepresented clones were identified (i.e., ST307, ST147, ST231, and ST13). However, ST307 was involved in a regional outbreak, whereas ST147 and ST13 were identified in different parts of France. Most of the patients colonized or infected with KPC-*Kp* had a clear link with Portugal, where these 4 STs were recently described to be the more prevalent (*30*,*31*). The KPC-2–producing *K. pneumoniae* isolates identified in France were predominantly recovered from patients with a history of travel in Greece (ST258) or Asia (ST11).

Regarding antimicrobial susceptibility of KPC-*Kp* in France, the relative high susceptibility to imipenem (30.3%) and meropenem to a lesser extent (18.2%) are in agreement with previous reports from Italy, where ST512 is highly prevalent (26.6% susceptibility to meropenem) (*32*). Conversely, data from the United States and Taiwan indicated that KPC-*Kp* are more resistant to carbapenems in those parts of the world, where ST258 is more prevalent (*33*,*34*).

Altogether, our results indicate that the KPC-*Kp* epidemiology has changed in Europe during the past 5 years. In 2018, ST258 and ST512 *K. pneumoniae* were no longer the main drivers of KPC resistance, at least in several non–KPC-endemic countries of western Europe, such as France and Portugal (*30*,*31*). KPC-*Kp* epidemiology also appears to have begun changing in some countries, such as Italy and Colombia, where CG-258 KPC-*Kp* was previously known to be endemic. This change is indicated by the reported emergence of ST307 and ST273 KPC-*Kp* in Sicilia (Italy) (*35*) and ST307 and ST14 KPC-*Kp* in Colombia (*36*). This change in the global epidemiology of KPC-*Kp* might have an effect on the identification of these carbapenemase producers with the molecular methods dedicated to the identification of GC258 *K. pneumoniae* (*37*,*38*).

In addition, our study highlights the dissemination of *bla*_KPC_ genes in high-risk clones of *K. pneumoniae* (ST307 and ST147), genetic features that might provide an advantage in adaptation to the hospital environment and the human host (*39*). These clones already convey several antimicrobial-resistance genes, including genes encoding other carbapenemases of NDM and OXA-48–like types (*40*,*41*). Accordingly, we might now fear the emergence of ST307 and ST147 high-risk clones of *K. pneumoniae* that can co-produce multiple carbapenemases. A recent study demonstrated the importance of ST307 in the dissemination of *bla*_OXA-181_ in South Africa (*42*). In that study, >600 isolates belonging to ST307 were recovered and analyzed, and the results demonstrated the importance of this clone as a carrier of carbapenemase genes in all continents. Another study used Bayesian analysis to demonstrate that ST307 emerged in the mid-1990s (*43*). ST307 had been strongly associated with the diffusion of *bla*_CTX-M-15_ (*43*) and now is associated with the dissemination of carbapenemase genes (*42*).

In conclusion, we found that the epidemiology of KPC-*Kp* has changed in Europe, in particular, with emergence of non–CG258 KPC-*Kp* isolates in France, linked to dissemination from Portugal. This change in epidemiology has to be considered by microbiologists because a few diagnostic assays specifically designed for the identification of ST-258 KPC-*Kp* isolates will not be able to detect non–CG258 KPC-*Kp* isolates.

Appendix 1 Antimicrobial resistance determinants of isolates in study of emergence of new non–clonal group 258 high-risk clones among *Klebsiella pneumoniae* carbapenemase–producing *K. pneumoniae* isolates, France.

Appendix 2 Phylogenetic analysis for study of emergence of new non–clonal group 258 high-risk clones among *Klebsiella pneumoniae* carbapenemase–producing *K. pneumoniae* isolates, France.
