# Supplementary material for: Emergence of New Non–Clonal Group 258 High-Risk Clones among Klebsiella pneumoniae Carbapenemase–Producing K. pneumoniae Isolates, France
Source: Emerg Infect Dis. 2020 Jun;26(6):1212–20. doi: 10.3201/eid2606.191517 (PMC7258464; doi:10.3201/eid2606.191517)
Supplement: Appendix 2 — Phylogenetic analysis for study of emergence of new non–clonal group 258 high-risk clones among Klebsiella pneumoniae carbapenemase–producing K. pneumoniae isolates, France. [file 19-1517-Techapp-s2.pdf]

Article DOI: https://doi.org/10.3201/eid2606.191517

Emergence of New Non–Clonal Group 258 High-Risk Clones among *Klebsiella pneumoniae* Carbapenemase–Producing *K. pneumoniae* Isolates, France

Appendix 2

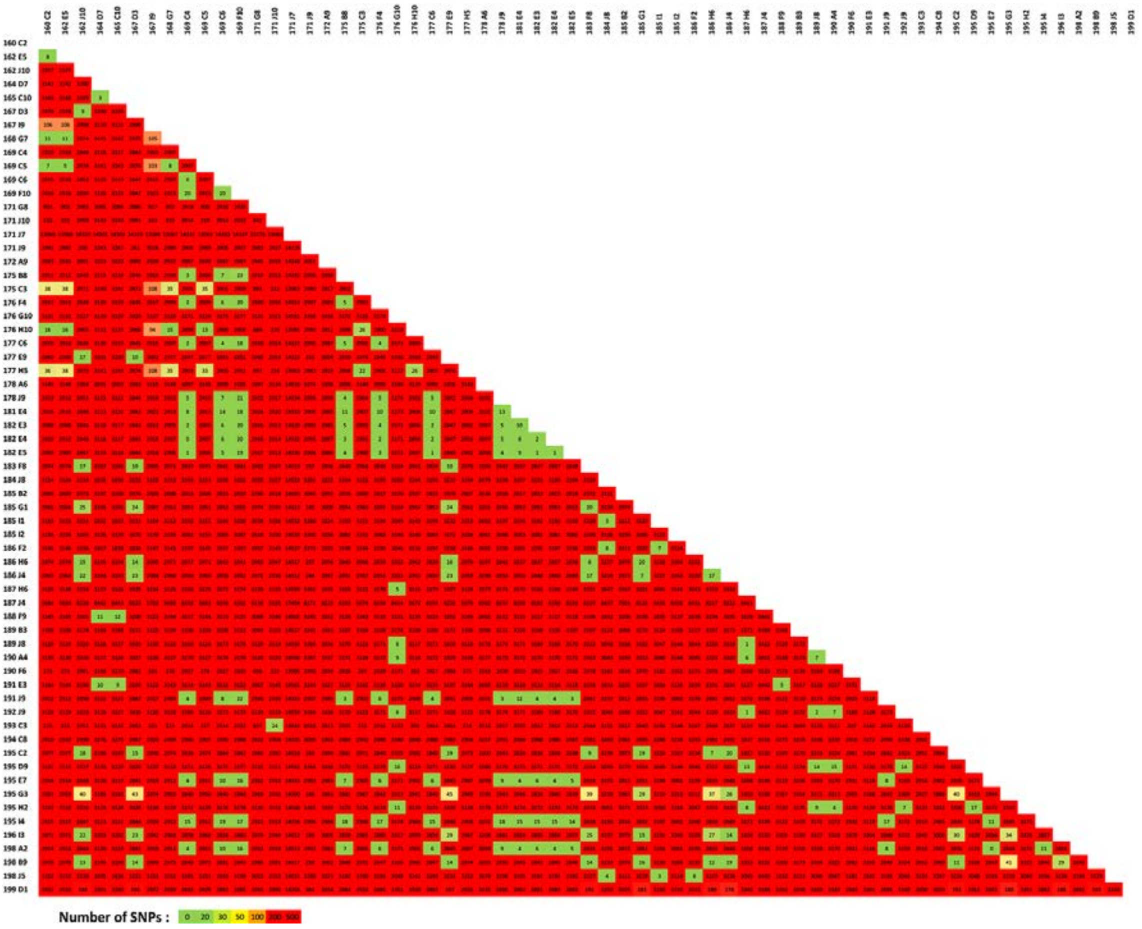

Appendix 2 Figure. Matrix of single-nucleotide polymorphisms (SNPs) between each KPC-producing *K. pneumoniae* isolate. Isolates with <21 SNP’s difference (dark green) are considered as identical.
